# The *bouba/kiki* effect is robust across cultures and writing systems

**DOI:** 10.1098/rstb.2020.0390

**Published:** 2022-01-03

**Authors:** Aleksandra Ćwiek, Susanne Fuchs, Christoph Draxler, Eva Liina Asu, Dan Dediu, Katri Hiovain, Shigeto Kawahara, Sofia Koutalidis, Manfred Krifka, Pärtel Lippus, Gary Lupyan, Grace E. Oh, Jing Paul, Caterina Petrone, Rachid Ridouane, Sabine Reiter, Nathalie Schümchen, Ádám Szalontai, Özlem Ünal-Logacev, Jochen Zeller, Marcus Perlman, Bodo Winter

**Affiliations:** ^1^ Leibniz-Zentrum Allgemeine Sprachwissenschaft, 10117 Berlin, Germany; ^2^ Institut für deutsche Sprache und Linguistik, Humboldt-Universität zu Berlin, 10099 Berlin, Germany; ^3^ Institute of Phonetics and Speech Processing, Ludwig Maximilian University, 80799 Munich, Germany; ^4^ Institute of Estonian and General Linguistics, University of Tartu, 50090 Tartu, Estonia; ^5^ Laboratoire Dynamique Du Langage UMR 5596, Université Lumière Lyon 2, 69363 Lyon, France; ^6^ Department of Digital Humanities, University of Helsinki, 00014 Helsinki, Finland; ^7^ The Institute of Cultural and Linguistic Studies, Keio University, Mita Minatoku, Tokyo 108-8345, Japan; ^8^ Faculty of Linguistics and Literary Studies, Bielefeld University, 33615 Bielefeld, Germany; ^9^ Department of Psychology, University of Wisconsin-Madison, Madison, WI 53706, USA; ^10^ Department of English Language and Literature, Konkuk University, Seoul 05029, South Korea; ^11^ Asian Studies Program, Agnes Scott College, Decatur, GA 30030, USA; ^12^ Aix-Marseille Université, CNRS, Laboratoire Parole et Langage, UMR 7309, 13100 Aix-en-Provence, France; ^13^ Laboratoire de Phonétique et Phonologie, UMR 7018, CNRS and Sorbonne Nouvelle, 75005 Paris, France; ^14^ Depto. de Polonês, Alemão e Letras Clássicas, Universidade Federal do Paraná, 80060-150 Curitiba, Brazil; ^15^ Department of Language and Communication, University of Southern Denmark, 5230 Odense, Denmark; ^16^ Department of Phonetics, Hungarian Research Centre for Linguistics, Budapest 1068, Hungary; ^17^ School of Health Sciences, Department of Speech and Language Therapy, Istanbul Medipol University, 34810 Istanbul, Turkey; ^18^ School of Arts, Linguistics Discipline, University of KwaZulu-Natal, Durban 4041, South Africa; ^19^ Department of English Language and Linguistics, University of Birmingham, Birmingham B15 2TT, UK

**Keywords:** perception, crossmodal association, iconicity, universals, sound symbolism

## Abstract

The *bouba/kiki* effect—the association of the nonce word *bouba* with a round shape and *kiki* with a spiky shape—is a type of correspondence between speech sounds and visual properties with potentially deep implications for the evolution of spoken language. However, there is debate over the robustness of the effect across cultures and the influence of orthography. We report an online experiment that tested the *bouba/kiki* effect across speakers of 25 languages representing nine language families and 10 writing systems. Overall, we found strong evidence for the effect across languages, with *bouba* eliciting more congruent responses than *kiki*. Participants who spoke languages with Roman scripts were only marginally more likely to show the effect, and analysis of the orthographic shape of the words in different scripts showed that the effect was no stronger for scripts that use rounder forms for *bouba* and spikier forms for *kiki*. These results confirm that the *bouba/kiki* phenomenon is rooted in crossmodal correspondence between aspects of the voice and visual shape, largely independent of orthography. They provide the strongest demonstration to date that the *bouba/kiki* effect is robust across cultures and writing systems.

This article is part of the theme issue ‘Voice modulation: from origin and mechanism to social impact (Part II)’.

## 1. Introduction

For decades, theoretical approaches and empirical data on the evolution of spoken languages have been dominated by the dogma of arbitrariness, according to which the forms of words do not resemble their meanings [1–3]. For example, there does not appear to be anything tree-like about the sounds of the English word *tree*. The fact that other languages have completely different forms for the same concept—such as German *Baum*, Spanish *arbor* or Chinese *shu*—suggests that form–meaning pairings are largely a matter of convention [4]. In line with the idea that arbitrariness prevails in spoken language, iconicity—the resemblance between form and meaning—has been thought to be largely confined to onomatopoeias, such as words like *bang* and *peep*, which imitate the sounds they denote.

In recent years, however, more and more research shows that such iconicity plays important roles in the evolution, acquisition and use of spoken language. First, growing evidence suggests that iconicity shapes the vocabularies of spoken languages far beyond the case of onomatopoeias. This is revealed, for example, through iconic form–meaning correspondences in basic vocabulary items [5–7], including terms for size [8,9], colour [6], textural properties [10], spatial deixis [11,12], shape [13] and more (e.g. [14,15]). On top of this, a growing number of experimental studies suggest the possibility that iconicity was important for the origins of spoken language [16,17], and may continue to shape the evolution of modern languages [18–21]. There also is evidence that iconicity performs important functions in language acquisition, with research showing that highly iconic words are easier to learn [22–25]. Moreover, iconicity has been shown to affect speech production [26–30]. For example, speakers have been shown to raise/lower their fundamental frequency when describing a small referent versus a big referent [28], or one that is positioned high versus low in space [29,31]. Together, this research has led to a dedicated shift in research, where iconicity is now recognized to be an important part of all languages [32,33].

Experimental research on iconicity in speech has often used pseudowords to probe what concepts certain sounds evoke. For example, in a classic experiment, Sapir [34] showed that English speakers matched pseudowords containing high front vowels, such as *mil*, to small objects; by contrast, they matched pseudowords with low back vowels, such as *mal*, to large objects. In the former case, the high fundamental frequency and high second formant frequency of the high front vowel /i/ is thought to give the impression of small size, given that small animals and objects generally produce higher-frequency sounds than large ones [35]. Hinton *et al*. [36] labelled these cases as *synaesthetic* sound symbolism to capture that the mapping between form and meaning cuts across sensory modalities with speech sounds representing content from other modalities, such as the visual or tactile properties of objects (e.g. shape, size). This connects the study of iconicity to the study of crossmodal correspondences [37–39], i.e. cases where participants reliably match stimuli across sensory domains. Iconicity in spoken languages, therefore, does not only involve the resemblance between speech sounds and auditory impressions but also resemblances between speech sounds and other sensory impressions that are mediated through crossmodal correspondences.

**Figure 1.**
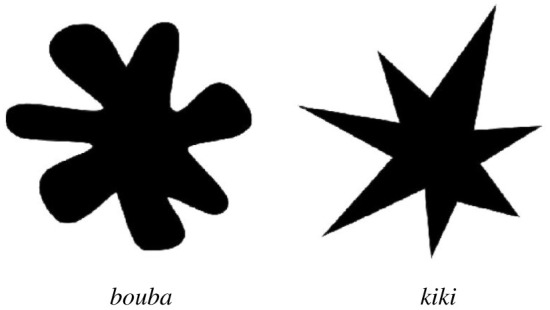
*Bouba* and *kiki* shapes used in the experiment (the names were not displayed in the online survey). The shapes were adapted from Bremner *et al*. [37].

Perhaps one of the most widely studied findings on the crossmodal associations evoked by speech sounds has been the so-called *bouba/kiki* effect. When asked to name the two shapes shown in figure 1 using the nonce words *bouba* and *kiki*, experiments indicate that the majority of participants will match *bouba* with the round shape and *kiki* with the spiky one. This general phenomenon was first demonstrated in Köhler's [40] work with two comparable words, *baluba* and *takete*, and in a later edition with *maluma* and *takete* [41]. The phenomenon was popularized in the twenty-first century by Ramachandran & Hubbard [42] with *bouba* and *kiki*. In each instance, people's matching behaviour demonstrates a correspondence across sensory modalities—between features of the visual shapes and features of the articulated sounds of the words. Ramachandran & Hubbard [42, p. 19] hypothesized that ‘the sharp changes in visual direction of the lines in the right-hand figure [see figure 1] mimics the sharp phonemic inflections of the sound kiki, as well as the sharp inflection of the tongue on the palate'. By virtue of this vocal mimicry—which renders a perceived resemblance between aspects of the spoken word and its meaning—the *bouba/kiki* effect is a prime example of iconicity in speech.

Ramachandran & Hubbard [42] proposed that the *bouba/kiki* effect and similar phenomena may provide a vital clue to the origins of language. They suggested that such intuitive mappings between aspects of the voice and certain visual properties would place ‘natural constraints on the ways in which sounds are mapped onto objects' [41, p. 19]. Making use of these perceived crossmodal correspondences, human ancestors might have modulated their voice to meaningfully draw attention to particular referents or qualities—a process that could have bootstrapped the formation of the first spoken words [23,43]. In the absence of stable conventions within a linguistic community, a word that is perceived to resemble its referent is more likely to be understood.

Notably, to the extent it is possible, this capacity to produce iconic signals with one's voice undercuts a key point of evidence in favour of accounts that see language as having first arisen on the basis of manual gestures. Often dubbed ‘gesture-first' theories of language origins, proponents of this hypothesis have long argued that manual gestures—which can be used to show spatial relationships, trace and depict shapes, and pantomime actions—have rich potential for iconicity, and thus they are especially useful for establishing meaningful communication when communicators lack a shared vocabulary (e.g. [44–46]). By contrast, it is argued that the voice does not offer the same iconic potential, being limited mainly to the mimicry of animal and environmental sounds and to the expression of emotion. For example, Hockett [47, p. 275] proposed that, ‘[w]hen a representation of some four-dimensional hunk of life has to be compressed into the single dimension of speech, most iconicity is necessarily squeezed out'. Indeed, if we restrict iconicity to onomatopoeia alone, the capacity for iconic expression in spoken language does appear to be quite limited. However, crossmodal correspondences such as the *bouba/kiki* effect can extend the role of spoken iconicity to include semantic domains that are not auditory in nature.

Stemming largely from its theoretical importance to questions of language evolution, the *bouba/kiki* effect has been replicated and extended numerous times in wide-ranging experiments, serving as a testbed for understanding the psychology of crossmodal correspondence in communication [48–55]. Moreover, while experiments using pseudowords have often been criticized for having limited relevance to spoken language vocabularies [56–58], recent evidence shows that the effect may actually influence the vocabularies of modern languages. Sidhu *et al*. [13] found that English nouns for round objects, such as *ball, globe, balloon* and *hoop*, are more likely to have round vowels and bilabial sounds than nouns for more angular or spiky objects, such as *spike, fork, cactus* and *shrapnel,* which are more likely to feature voiceless velar stops. Thus, in the same way that Sapir's [34] *mil*/*mal* experiment corresponds to iconicity for magnitude in vocabularies [5,8,9], there now is evidence that *bouba/kiki* is not confined to artificially created pseudowords. The potential for *bouba/kiki* to play a role in language evolution has now also been lifted from the realm of speculation thanks to new empirical evidence from iterated learning experiments [20,21]. This work shows that when generations of participants learn and reproduce an artificial spoken vocabulary that refers to visual stimuli such as those shown in figure 1, the word forms that they produce evolve over iterations to exhibit *bouba/kiki-*like iconicity. Taken together, this research shows that crossmodal sound-symbolic mappings such as in *bouba/kiki* could play an ongoing role in the development of spoken language vocabularies. However, there remains controversy regarding the source of the *bouba/kiki* effect, and the extent to which the mapping between sound and shape is consistent across cultures.

## 2. Source of the *bouba/kiki* effect

Since Köhler's preliminary experiments, a primary line of inquiry has investigated the specific sources of the *bouba/kiki* effect—that is, the crossmodal correspondences between auditory voice and visible shape that are involved. A number of vocal cues have been proposed, which may relate to both the acoustics of the speech sounds and the proprioception of articulating them [38,59]. The pseudowords *bouba* and *kiki*—and comparable forms like *maluma* and *takete*—differ from each other along a number of phonetic/phonological dimensions [48,52,60,61], yielding strikingly different acoustic and articulatory profiles. These differences relate to vowel formants, vowel-intrinsic fundamental frequency, consonant-driven fundamental frequency perturbation, duration, consonant voicing, voice onset time, vowel rounding and place of articulation, all of which can influence the *bouba/kiki* effect in some fashion or another [13,59,60,62,63]. For example, voiced stops [60], round vowels and labial consonants are associated with round shapes [13,60]. The effect may also be owing to broader patterns in the spectral envelopes of the words [54]. Abrupt spectral changes from silent closure to high spectral frequencies caused by voiceless stops may relate to spikiness in the visual domain. By contrast, continuous fundamental frequency, as is present in words such as *bouba* and *maluma*, goes together with lower frequency bands and less abrupt amplitude envelope modulations, which may evoke a sense of smoothness in perception, relating this to visually smoother, or rounder objects. There are, therefore, clear ways in which phonetic/phonological characteristics of *bouba* and *kiki* may be associated with round and angular shapes, respectively [64].

There is also reason to believe, however, that these phonetic/phonological explanations do not provide a complete account of the source of the effect. One can readily see that the letters of the Roman alphabet used to represent *kiki* 〈k, i〉 are visually spikier than the more rounded letters for *bouba* 〈b, o, u, a〉, and the same also characterizes the contrast between words like *takete* and *maluma*. This raises the possibility that many experiments demonstrating the *bouba/kiki* phenomenon—the majority of which have been conducted with literate Western participants—may be confounded by the orthographic shape of the written words [65–68]. Importantly, because writing is a highly entrenched cognitive process, orthographic representations can become automatically activated even in completely auditory tasks [69–71]. This renders it plausible that orthography may be a confound even when pseudowords are presented auditorily.

Evidence of this confound comes from Cuskley *et al*. [65], which found that grapheme shape was a dominant source of the effect for literate, English-speaking participants. Participants were asked to rate the goodness of fit of different pseudowords with various rounded and spiky shapes, with the word presented in either written or auditory form. Critically, the stimuli varied in phonemic characteristics (i.e. voicing contrast) as well as orthographic angularity, allowing the effects of these variables to be separated. The results showed that orthographic angularity was the ruling factor in the written task with no effect of phonology, whereas both orthographic angularity and phonology played a significant role in the auditory task. Notably, such an orthographic confound may also extend beyond just Roman orthorgraphy. Turoman & Styles [72] presented—to an international group of English speakers—pairs of letters representing the speech sounds /u/ and /i/ (i.e. the stressed vowels in *bouba* and *kiki*) in 56 different scripts from across historical time and geographical space. They found that participants were significantly better than chance at guessing which sound each letter represented. These studies show that orthography rather than crossmodal correspondences may drive the *bouba*/*kiki* effect—in Roman orthography as well as many other scripts.

While orthography can clearly play a role in the *bouba/kiki* phenomenon, evidence from studies with different populations suggests that there is also some genuinely vocal basis for the effect. For example, early blind individuals who have no experience with the Roman alphabet show the effect when feeling round and pointy shapes [73], although earlier investigations failed to establish this [74]. While a few studies have failed to find the *bouba/kiki* effect with pre-literate children [75,76], several others have shown the effect in children, including pre-literate ones [51,77–79]. However, given that sound-symbolic phenomena such as *bouba/kiki* generally become stronger with age [34,78,80–82], more convincing evidence for the idea that orthography is not the locus of *bouba/kiki* comes from cross-cultural studies with speakers of non-literate societies. For example, speakers of Himba (a Bantu language spoken in Namibia) showed the *bouba/kiki* effect even though they were non-literate and had minimal exposure to Western culture [37]. Another study found that Taiwanese participants showed similar *bouba/kiki* performance to United States participants, despite the fact that their languages are written in different scripts [83]. More generally, a meta-analysis of 13 different *bouba/kiki* experiments with speakers of six different languages (English, French, Italian, Himba, Syuba and Hunjara) showed that across languages, 89% of all responses were congruent with the phenomenon [84]. However, some exceptions have also been found: Syuba speakers from the Himalayas in Nepal did not show the effect [84], and neither did Hunjara speakers in Papua New Guinea [85]. Styles & Gawne [84] suggested that the lack of effect for these two groups may be because the nonce words have some phonemes that do not occur in the respective language or that some sounds are phonotactically/tonotactically illegal in the corresponding languages.

In this paper, we report, to our knowledge, the most extensive experimental test of *bouba/kiki* to date, using a diverse sample of speakers from 25 different languages and nine different language families that use a total of 10 different scripts. Our diverse sample of participants allowed us to use variation in orthographic systems as a testbed to perform a natural experiment on the influence of writing systems on the *bouba/kiki* effect across languages. While previous meta-analyses of cross-linguistic data suggest that *bouba/kiki* may be cross-linguistically stable [84]—with some notable exceptions—our experiment tests the phenomenon under exactly the same experimental conditions. Demonstrating that *bouba/kiki* exists across speakers of multiple languages and is not strongly affected by writing systems would show that the effect is based on a genuine crossmodal correspondence between sound and shape. Moreover, by demonstrating cross-cultural stability and relative independence from orthography, the phenomenon takes on greater relevance for theories of the origins of spoken language.

In contrast to other investigations that have explored a range of pseudowords with different phonological properties or a range of different visual stimuli [52,59–61,86], we followed Bremner and colleagues' investigation of Himba speakers [37] and focused on the specific pseudowords *bouba* and *kiki*. In using these two stimuli across a large opportunity sample of languages, we increased our statistical power to generalize across languages and cultures. For this, we used standard statistical methods from linguistic typology, namely, mixed-effects models with random effects for language family (e.g. [87–89]), which help us to avoid ‘Galton's problem' of erroneously treating different languages/cultures as independent [90,91]. Thus, our goal was not to demonstrate that *bouba/kiki* is universally obeyed by all speaking populations in an absolute sense, i.e. that speakers from each and every language exhibit the phenomenon (cf. [92]). We expected exceptions, and given that our data collection method involved an opportunity sample with unequal data points for different language groups, our goal was also not to make precise claims about speakers from specific cultural groups. Rather, our aim was to assess the extent to which the *bouba/kiki* effect—and the crossmodal sound-shape correspondence that underlies it—is a widespread cross-cultural phenomenon.

## 3. Methods

### (a) Participants

We collected data from a total of 976 participants. This was an opportunity sample, with data collected via snowballing. Participants were recruited by contacting native speakers of each language and asking them to distribute the survey among other native speakers. The distribution of the questionnaires took place between August and December 2018. We aimed to obtain at least 20 participants per language, but this proved not to be possible for several languages. Sample size decisions were made independently of the results. Participation was voluntary and self-motivated, and participants received no compensation (with the exception of Zulu speakers).

Data were then excluded from participants who did not complete both the *bouba* and the *kiki* trials or who indicated that they did not speak the language of the respective survey. Data from speakers of two languages—Malagasy and Tamil—were excluded because we had only one and two participants respectively for these groups, meaning that survey distribution effectively failed.^1^ Using these criteria together, a total of 59 participants (6%) were excluded. The remaining sample contained data from 917 participants of 25 languages from nine different language families, shown in table 1. The 25 languages included 10 different scripts. The Roman script was shared by many different languages (e.g. English, German, French). On top of that, there were nine languages that dominantly use scripts other than the Roman script: Armenian, Farsi, Georgian, Greek, Japanese, Korean, Mandarin Chinese, Russian and Thai.

**Table 1.** Counts of participants (and *bouba*-first trials) per language and language family, ordered alphabetically by language name within family and genus (based on [93]); Italics-faced languages marked by * dominantly use scripts without Roman letters.

family	genus	language	*n* of participants (*n* of *bouba*-first trials)
Indo-European	Albanian	Albanian	10 (6)
	Armenian	Armenian*	22 (13)
	Germanic	Danish	18 (10)
		English	41 (16)
		German	87 (45)
		Swedish	21 (13)
	Greek	Greek	40 (19)
	Iranian	Farsi*	22 (13)
	Romance	French	57 (25)
		Italian	54 (33)
		Portuguese	59 (30)
		Romanian	33 (16)
		Spanish	35 (21)
	Slavic	Polish	52 (26)
		Russian*	49 (25)
Japanese	Japanese	Japanese*	55 (35)
Kartvelian	Kartvelian	Georgian*	14 (8)
Korean	Korean	Korean*	22 (13)
Atlantic-Congo	Bantu	Zulu	20 (10)
Sino-Tibetan	Chinese	Mandarin Chinese*	49 (23)
Tai-Kadai	Kam-Tai	Thai*	20 (8)
Turkic	Turkic	Turkish	38 (18)
Uralic	Finnic	Estonian	46 (27)
		Finnish	19 (11)
	Ugric	Hungarian	35 (19)

Our survey asked participants to report any foreign languages that they speak. Eighty six per cent of our participants indicated they spoke a second language, with 80% speaking English as a first (L1) or second language (L2). This left 179 participants who did not speak English. Of the 293 participants who did not speak a first language with a Roman script, our sample included 55 participants who also did not speak a second language with a Roman script. As noted by Grosjean [94], at least half of the world's population speaks more than one language, and a more recent survey of countries of the European Union [95] found that on average 63% people speak another language, ranging from 74% in people aged 25 to 34 years to 47% in people aged 55 to 64 years. Thus, our sample has a relatively large percentage of people speaking a foreign language, especially English. The large percentage of speakers of English as a foreign language could stem from the fact that the sample was snowballed using the authors' own social networks. Moreover, the internet-based distribution method probably taps into a more educated population.

### (b) Materials

**Figure 2.**
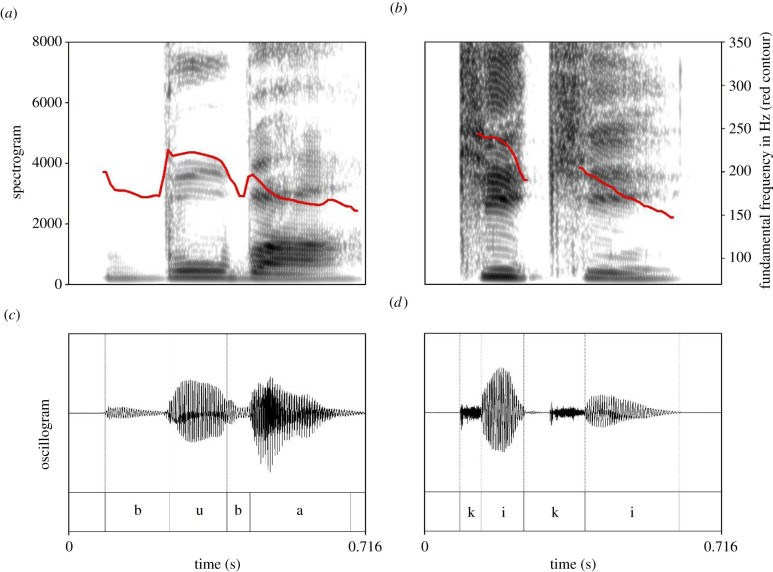
Spectrograms (*a,b*) with fundamental frequency marked as a red contour and oscillograms (*c*,*d*) of the pseudowords *bouba* (*a*,*c*) and *kiki* (*b*,*d*).

The rounded and spike shapes, shown in figure 1, were adapted from the cross-cultural experiment conducted by Bremner *et al.* [37]. The *bouba* and *kiki* stimuli were spoken by the first author, a female native speaker of Polish who is a trained phonetician. *Bouba* was rendered as [′bu:ba], and *kiki* as [′k^h^ik^h^i], both with initial stress. Figure 2 shows spectrograms and oscillograms of the recording for each word. The stimuli are accessible via the following Open Science Framework (OSF) repository: https://osf.io/w7crs.

### (c) Procedure

Our data were collected as part of a larger cross-linguistic computer survey administered online using the Percy software [96]. The *bouba/kiki* task was completed at the end of the roughly 15 min survey, whose results are reported elsewhere [16]. Participants viewed the rounded and spiky shapes as they listened to the spoken words *bouba* and *kiki* in two successive trials. The stimuli were presented to each participant in random order (resulting in 52.5% of participants listening to *bouba* first; table 1). On each trial, after listening to the word, they selected which of the two shapes they thought better corresponded to the word (a forced-choice task). Participants were instructed to look at the two shapes and to listen to the sound. They were then asked: ‘which shape corresponds to the sound?' This approach differs from the frequently used two-alternative forced-choice task, which has been shown to amplify the *bouba/kiki* effect [52,57,86].

The survey was distributed in 25 different languages, translated by native speakers of the respective languages. The languages were chosen to facilitate a sample as diverse as possible based on the availability of researchers able and willing to collaborate on the project.

### (d) Analysis

Our main analysis examined the proportion of *bouba/kiki*-congruent responses across both trials. For this, we counted only those participants who matched *bouba* to the round shape, and crucially, who also matched *kiki* to the spiky shape. If one of the two trials was misaligned with *bouba/kiki*, the response was treated as incongruent. This measure of the effect thus treats partial matches as mismatches. By treating the data like this, the individual participant becomes the unit of analysis (each participant contributes one data point). To determine the overall strength of the effect across languages, we fitted a mixed Bayesian logistic regression model; the intercept of this model estimates the average proportion of participants who responded with a ‘matching' word-to-shape alignment. This proportion was compared to a conservative baseline of one-half = 50%, assuming complete dependence of the two trials (even though chance-level would be one-quarter = 25% if the two trials were treated as independent). The model also included two fixed effects: order (whether *bouba* or *kiki* was the first trial) and script (whether the language predominantly uses the Roman alphabet or a different script). As the order-fixed effect was effectively balanced (approximately half of the trials were *kiki-*first; half were *bouba-*first), we contrast-coded *kiki* as −0.5 and *bouba* as +0.5. For script, our sample was biased towards the Roman alphabet, and, therefore, we used weighted effect coding to adjust for sample size differences (+1 for Roman alphabet, −2.12 for different alphabet). This contrast coding scheme of our fixed effects ensured that the intercept was interpretable as the grand mean.

We included random intercepts for language and language family. The language isolates Japanese and Korean were treated as separate families. In addition, we added random slopes for within-language and within-family variation in the order effect. We did not add random slopes for the script effect, because only the Indo-European languages showed variation with respect to the script factor. All other language families in our sample were of only one script. We set *Normal*(*0, 1*)** weakly informative priors on the intercept. This prior choice was guided by recommendations for logistic regression models in Lemoine [97]. Under a logit transform, the prior builds in mild skepticism, slightly favouring values closer to chance performance (*p* = 0.5) and punishing high values close to 0 or 1. For all beta coefficients, we use a Cauchy prior with scale = 2.5 [98]. The model was estimated via Markov chain Monte Carlo sampling with four chains, each with 6000 samples (of which a total of 4000 were discarded as warm-up samples), making for a total of 8000 posterior samples used for inference. All data and code are accessible via the following OSF repository: https://osf.io/w7crs.

### (e) Additional analysis of orthography

We also investigated the more general possibility that a bias in the orthographic shape of the words in the respective writing scripts—that is, beyond the known bias of the Roman script—could drive the *bouba/kiki* effect across cultures. To assess the orthographic bias for each script used by our participants, we conducted a subsidiary study to generate a measure of how much each script is biased towards *bouba/kiki* congruency. We asked an independent set of participants to match the written representation of *bouba* and *kiki* in each script to the corresponding rounded/spiky shapes based only on visual similarity. Participants were instructed to match each bit of text ‘to the shape that you think looks most similar to the text.' It was emphasized that they should focus only on the visual appearance of the text and shapes.

While it is possible to compute the visual similarity between letters via objective computational techniques [99], we decided to use a behavioural measure of orthography bias because what arguably matters more for the *bouba/kiki* paradigm is the *perceived,* rather than physical, spikiness and roundedness of shapes. Any computational measure would have to be independently calibrated via additional studies to assure that it taps into people's perceptions of spikiness/roundedness.

For this subsidiary study, we recruited German participants (*n* = 97) and Mandarin Chinese participants (*n* = 78) via snowballing, and English participants (*n* = 78) via Amazon Mechanical Turk (table 2). Each participant viewed the words *bouba* and *kiki* written in each of the nine non-Roman scripts. On each trial, a written word was presented above the two shapes, which were presented beside each other. Participants then selected one shape or the other. Trials were paired by language, and in analogy to our main experiment, the written stimuli were shown sequentially. We randomized the order of languages, the order of *bouba/kiki*, and the order of the shapes (rounded-left or spiky-left). After completing the task, we asked participants to report any knowledge of the corresponding scripts or languages they saw, excluding any trials for which this was the case. This led to the exclusion of 10% of the total trials, from 3538 individual trials down to 3192.

**Table 2.** Orthographic representations of *bouba* and *kiki* written in the scripts of the languages that were included in the survey; the additional columns show the percentage of *bouba/kiki* congruent matches for each script by speakers of English, German and Mandarin Chinese. The average of the three language groups was used as a predictor in the main experiment (the auditory task).

language	bouba	kiki	English (*n* = 51)	German (*n* = 97)	Mandarin Chinese (*n* = 78)	average
Armenian	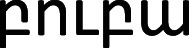	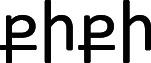	45%	56%	59%	53%
Cyrillic script	буба	кики	74%	82%	71%	76%
Farsi	بوبا	کیکی	48%	44%	51%	48%
Georgian	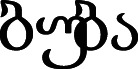	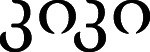	50%	48%	48%	49%
Greek	μπούμπα	κίκι	69%	70%	70%	70%
Hangul script (Korean)	부바	키키	43%	49%	71%	54%
Japanese (Katakana)	ブーバ	キキ	49%	52%	62%	54%
Mandarin Chinese	布巴	奇奇	58%	56%	—	57%
Roman script	bouba	kiki	—	—	—	—
Thai	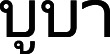	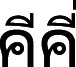	51%	44%	41%	45%
average			54%	55%	59%	56%

To determine the effect of orthographic bias in the main (auditory) experiment, we averaged the proportions of congruent responses across the three different languages to get an aggregate measure of orthography bias.^2^ These average values were included as a predictor into an additional model, where we regressed the proportion of *bouba/kiki* congruent responses in the auditory task on the orthography bias score from the subsidiary study.

## 4. Results

### (a) *Bouba/kiki* effect across languages

**Figure 3.**
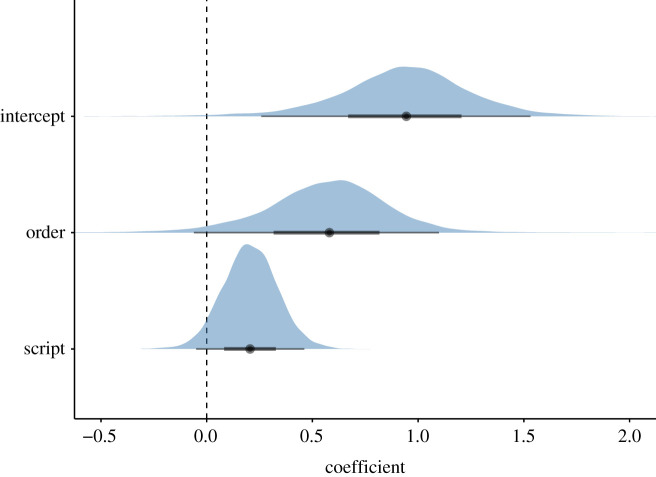
Posterior distributions of the coefficients from the main model; contrast coding for order predictor: *kiki-*first = −0.5, *bouba-first* = + 0.5; weighted effect coding for script predictor: other script = −2.12, Roman = +1; horizontal black lines show the 95% credible interval; thick boxes the 50% interval; points show the median. (Online version in colour.)

The estimated posterior mean proportion of *bouba/kiki* matches across languages was 72%, with a 95% credible interval ranging from 56% to 82%. The logit coefficient of the intercept was above zero (+0.93, s.e. = 0.31), with the 95% credible interval not including zero: [+0.26, +1.53]. The posterior distribution of this coefficient is shown in figure 3 (top). The estimated posterior probability of the logit intercept being above zero (=chance level) was very high ($p(\beta_{0}>0)=0.99$).

**Figure 4.**
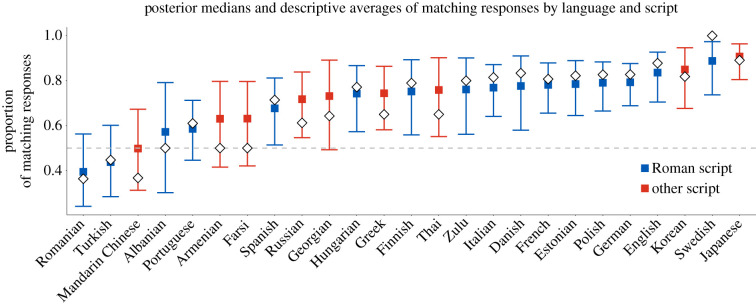
Posterior medians (coloured squares) of the proportion of matching responses (*bouba* = round shape; *kiki* = spiky shape) for each language with the corresponding 95% credible intervals (coloured vertical segments) from the Bayesian logistic regression reported in the body of the text; white diamonds indicate the raw descriptive averages; languages are ordered by increasing posterior means; the grey dashed line shows the baseline level (=50%). (Online version in colour.)

When analysed by individual languages, the descriptive percentages ranged from 100% (Swedish) to 36% (Romanian). Figure 4 shows the posterior estimates for each language with corresponding 95% credible intervals. Using these credible intervals as a heuristic cut-off point, 17 out of 25 languages showed a *bouba/kiki* effect that was reliably above 50%. However, it should be noted that this measure is rather conservative, as, for most languages, the bulk of the posterior distribution was above zero. In terms of descriptive averages, only three languages—Romanian, Mandarin Chinese and Turkish—had lower than 50% matches though the 95% credible intervals included chance level performance.

**Figure 5.**
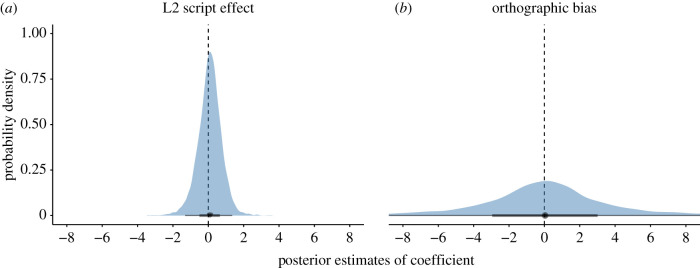
(*a*) Posterior samples for the L2 script effect (model that excludes participants who use a Roman alphabet in their L1); (*b*) posterior samples for the orthographic bias score (model on non-Roman script languages only, excluding Greek and Russian). (Online version in colour.)

Languages that predominantly use the Roman script had numerically higher *bouba/kiki* matches (descriptive average: 75%) than languages that use other scripts (63%). The model indicated that there was a weak trend for languages with the Roman script (versus other) to have a higher proportion of matches (logit coefficient: +0.21, s.e. = 0.13), the posterior estimate of which is shown in figure 3 (bottom panel). The 95% credible interval for this coefficient overlapped with zero: [−0.05, +0.46]. The posterior probability of the script effect being above zero was relatively high ($p(\beta_{0}>0)=0.95$), but much less so than the posterior probability of the overall accuracy effect being above zero. In addition, for those participants who did not speak a language with the Roman script as an L1, we analysed whether speaking a language as an L2 that did not use the Roman script changed the proportion of *bouba/kiki* matches. There was no evidence that this was the case, with the coefficient of the L2 script effect close to zero (+0.08, s.e. = 0.66), and its wide 95% credible interval being centred on zero: [−1.31, +1.35] (figure 5*a*).

### (b) Additional analysis of script

Next, we turn to our perceptual measure of orthography bias from the subsidiary study. Across the nine non-Roman scripts, participants, on average, matched the written words to the corresponding shapes in a manner that was congruent with the *bouba/kiki* phenomenon 56% of the time (English: 54%, German: 55%, Mandarin Chinese: 59%). As shown in table 2, there is clear variation in the orthographic shape bias across scripts, ranging from the lowest of Thai (45%) to the highest of Greek (70%) and Cyrillic (76%). The high scores for Greek and Cyrillic are perhaps unsurprising given that the scripts are related to the Roman alphabet. Moreover, the orthographic renditions of the pseudowords *bouba* and *kiki* in these scripts share some of the same characters as the orthographic renditions in the Roman alphabet. The results were fairly similar between the three language groups. English and German speakers' average proportion of congruent responses correlated very highly (*r* = 0.88), as did German and Mandarin Chinese speakers' average (*r* = 0.7). The correlation was still positive but weaker between English and Mandarin Chinese (*r* = 0.43).

To analyse the effect of this orthographic perceptual bias, we used the average proportion of congruent orthography responses from across the three languages as a predictor of *bouba/kiki* matches in the auditory experiment. For this Bayesian regression model, we only considered the subset of participants who spoke languages with non-Roman scripts (*n* = 293). The coefficient of the orthography bias measure was positive (logit: +0.74, s.e. = 1.90), but associated with an exceedingly large 95% credible interval, [−3.17, +4.41], that included zero, as shown in figure 5*b*. The posterior probability of the effect being positive was inconclusive ($p(\beta_{1}>0)=0.68$), suggesting that this analysis does not indicate a reliable effect of the orthography bias measure. This is the case even if we exclude Greek and Russian from the analysis, given that these two scripts are similar to the Roman alphabet ($p(\beta_{1}>0)=0.51$).

### (c) Is *bouba* more round than *kiki* is spiky?

**Figure 6.**
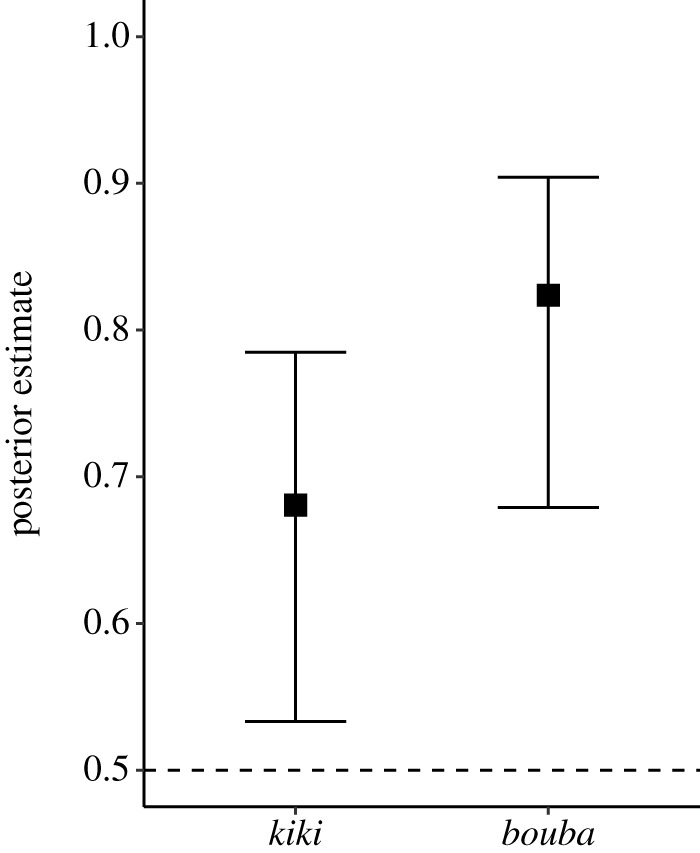
Analysis of first trials only shows that *bouba* trials were more accurate than *kiki* trials; black boxes indicate posterior medians; error bars indicate 95% credible intervals.

The main model reported above included an effect of order (figure 3, middle). The logit coefficient of this order effect was positive (+0.56, s.e. = 0.29) indicating that matching responses were produced somewhat more often for *bouba*-first trials than for *kiki*-first trials (95% credible interval: [−0.06, +1.10]). The posterior probability of this coefficient being positive was relatively high ($p(\beta_{1}>0)=0.97$). Recall that for these results based on our main model, we considered a ‘match' conservatively as cases where *both* trials fit the *bouba/kiki* effect. To better assess the asymmetry between *bouba* and *kiki* trials, we looked at first trials only in a separate model with a fixed effect for condition (whether the first trial was *bouba* or *kiki*). The logit coefficient of condition was positive (more matches for *bouba*: +0.79, s.e. = 0.34), with a 95% credible interval that did not cover zero: [0.08, 1.45]. Figure 6 visualizes the conditional effects.

Finally, we used the model fitted to first trials only to assess the number of languages for which *bouba* trials were above chance, compared to *kiki* trials. The corresponding 95% credible intervals for each language showed that *bouba* was reliably matched with the round shape in 22 out of 25 languages, whereas *kiki* was reliably matched to the spiky shape in only 11 out of 25 languages.

## 5. Discussion

We tested whether people from diverse cultural and linguistic backgrounds exhibited the *bouba/kiki* effect, matching the nonce word *bouba* to a round shape and *kiki* to a spiky one. Our internet survey reached 917 participants who were speakers of 25 languages from nine language families, including 10 different writing systems. We found strong overall evidence for the effect across participants, demonstrating a clear cross-linguistic pattern. Notably, we demonstrated the cross-cultural consistency of the *bouba/kiki* effect using a more conservative measure of the phenomenon, requiring participants to produce congruent matches for both *bouba* and *kiki* trials, and setting the comparison to chance level at 50% rather than 25%. In comparison to sequential presentation, it has been suggested that the two-alternative forced-choice task, where both nonce words and both shapes are presented simultaneously, may artificially amplify the effect size [52,57,86]. For example, when the stimulus images are presented sequentially rather than simultaneously, the proportion of congruent responses dropped from 80% to 60% in [52]. Here, we found the overall proportion of congruent responses to be 72%, indicating that the cross-cultural effect is robust even in a sequential presentation format.

We also found *some* evidence consistent with the idea that Roman orthography can play a role in enhancing the *bouba/kiki* effect [65]: languages that predominantly used the Roman alphabet showed a stronger effect than those that did not. Overall, however, the biasing effect of orthography was weak. Participants who used a script other than the Roman alphabet were, on average, far above chance (63%) in producing congruent responses. This was the case even given our more conservative measure of full matches across both trials. Moreover, for those participants who did not use the Roman alphabet in their L1, speaking a second language that used the Roman alphabet did not make the *bouba/kiki* effect stronger. Finally, our subsidiary experiment showed that the degree to which the forms of the words written in non-Roman scripts were perceived to be rounder for *bouba* and spikier for *kiki* also did not alter the results.

One qualification in interpreting our results is that most of our participants spoke a foreign language (with English being the modal foreign language), and most participants knew a language that used the Roman alphabet. Even those participants who did not report speaking a language that used the Roman alphabet would probably have had some experience with this script given our use of an internet-based survey, e.g. Chinese speakers use Roman letters (Pinyin) when interacting with computers. From this perspective, then, it is important to see our results alongside the fact that the *bouba/kiki* effect has also been found in congenitally blind individuals [73], in at least some studies of pre-literate children [51,77,79], as well as in some cultures that do not use written language [37]. Taken together, this evidence converges to indicate that *bouba/kiki* exists, to a substantial extent, independently of orthography, and thus, appears to be rooted in crossmodal correspondence between the spoken words and visual shapes. However, as observed by [65,72], this does not imply that the sound-symbolic correspondence and the orthographic shapes connected to those sounds are not deeply intertwined—in cultural evolution, as well as in perceptual processing. Indeed, congruent orthographic shapes for the sounds /i/ and /u/ in the Roman alphabet and other scripts could be rooted historically in something like the sound symbolism of *bouba-kiki*, today feeding back into people's behaviour in modern *bouba-kiki* experiments.

As we have only tested two pseudowords rather than a whole set of words systematically varying in their phonetic properties, we can only speculate about the sound-symbolic root of the effect. As purely acoustic objects, we suggest that the continued presence of voicing in *bouba* may play an important role, especially as voicing has been shown to have drastic effects on the overall amplitude and spectral characteristics of spoken utterances [100]. Portions including phonation have a lower average centre of gravity (a global measure of frequencies with high amplitude) compared to portions without phonation. Thus, as shown in figure 2, this means that *bouba* has an overall lower spectral energy than *kiki*. Although acoustically, spectral energy is different from fundamental frequency, people mentally associate high spectral loci with high pitch [101]. Given previous findings indicating that low pitch is associated with roundness [102], this suggests that the overall spectral energy being lower or higher—as a result of the consistent phonetic voicing difference between the stimuli—may contribute to the effect. In addition, the intermittency of voicing in vowels and voicelessness in the aspirated stops in *kiki* leads to clearly visible spectral discontinuities (figure 2), which suggests that the overall smoothness or abruptness of the sound may also be an important factor (cf. discussion in [54]).

The sequential presentation format of our experiment allowed us to compare the strengh of *bouba* to *kiki* correspondence in a large cross-linguistic sample. The comparison of *bouba*-first to *kiki*-first trials showed that, although congruent responses to both words were well above chance, *bouba* evoked a stronger shape correspondence than *kiki*. This is in line with several studies which have now found that *bouba* produces more consistent matching behaviour than *kiki* [103,104], including a meta-analysis of language acquisition studies involving *bouba/kiki* [78]. One possible explanation for this asymmetry relates to the phonological properties of the words: *bouba* [′bu:ba] arguably uses more of the acoustic cues associated with round shapes than does *kiki* [′k^h^ik^h^i] with spiky shapes [13,48,60,62]: /b/ is a voiced bilabial stop, and /u:/ is rounded. All of these features (labial place of articulations, voicing and lip rounding) have independently been shown to be associated with round shapes [13,48,60,62]. Moreover, all segments of the word *bouba* are phonologically voiced, which is also visible phonetically in the entire word by the continuous fundamental frequency in figure 2. In comparison, *kiki* exhibits alternation between phonologically voiced segments (vowels) and voiceless segments (plosives), phonetically visible as an interruption of fundamental frequency. The word *kiki,* by virtue of combining phonologically voiced and voiceless sounds, can be seen as involving conflicting cues for spikiness [60], at least with respect to the phonetic dimension of voicing, ergo yielding a weaker effect, as our results show. An additional possibility is that our results reflect a general visual preference for curved contours, which has been shown in humans [105] as well as great apes [106]. This could have the effect of amplifying congruent responses in *bouba* trials and decreasing congruent responses in *kiki* trials, independently of perceptual correspondence between word and shape.

An interesting avenue for future exploration is whether the *bouba/kiki* effect is expressed in iconic prosody. We already know that during speaking, people sometimes modulate their voice in iconic correspondence with visual characteristics such as vertical position [29,31,107], motion [30,108] and size [28]. Would speakers similarly modulate their voice during speaking to express shape? A specific prediction is that speakers should enhance their glottal vibration (low frequency energy) when talking about round objects than talking about angular objects. They may also emphasize high frequency energy (by using stronger bursts) when talking about angular objects. Notably, such iconic modulations could, over historical time, lead to sound-symbolic vocabulary, including, for example, the statistical tendency for *bouba/kiki* sound symbolism in English nouns [13].

Future research is necessary to understand why speakers of a few languages tended not to show the common *bouba/kiki* alignment. Although the minority in our sample, these exceptions spanned languages spoken by huge populations (Mandarin Chinese), and languages with Roman scripts (Albanian, Turkish, Romanian). Possible factors to explain the absence of the effect in these languages include lack of specific phonemes, the tono- or phonotactics of the language [84], or the existence of meaningful words that happen to sound like *bouba* or *kiki* [72]. For example, in the case of Romanian, which had the lowest proportion of correspondence, the word *bouba* could be seen as related to the Romanian word *bubă* [bubə], a generic term for ‘wound' (including cuts and burns), used especially with small children. It may be that the association with sharp pain overrides the tendency to associate *bouba* with a round shape.

Whatever explains the exceptions in each specific case, our data clearly allow the conclusion that the *bouba/kiki* phenomenon is statistically robust across cultures. The strength of the *bouba/kiki* phenomenon is modulated by linguistic and cultural background, but there is a strong overall universal trend in this crossmodal correspondence. This has important implications for the evolution of language. If *bouba/kiki* were exclusively tied to writing systems and only observed for specific language groups, it could not have played any role in the origins of spoken language. By demonstrating that a correspondence between vocal signals and visual shapes is widely recognized irrespective of writing systems, *bouba/kiki* becomes more relevant for theories of language evolution [20,21,41,43]. It suggests that crossmodal correspondences such as *bouba/kiki* could have been used to extend iconicity in spoken languages beyond onomatopoeia, to include such domains as shape (as investigated here), but also size, touch and colour properties, and potentially many others. Our results are thus broadly in line with evidence from tasks where participants communicate meanings with novel vocalizations, which show that iconicity in vocalization enables understanding in the absence of established linguistic conventions [16,28,109].

Not withstanding the limitations discussed above, our study provides, to our knowledge, the strongest demonstration to date that the *bouba/kiki* effect extends across cultures. Our cross-linguistic survey included participants who were speakers of diverse languages spanning several language families and writing systems. The overall consistency of the phenomenon suggests that the effect is rooted in a robust crossmodal correspondence between speech sounds and visual shapes. Thus, while language, script and other elements of culture may play a mediating role in the strength of the effect, there is, nevertheless, a strong tendency for people across the globe to associate the spoken word *bouba* with a round shape and *kiki* with a spiky one.

## Endnotes

1 While it is possible to include these languages and count them towards the global average in the statistical analysis, we decided not to do so in line with our reasoning that the results of individual languages should not be over-interpreted. For these languages, we have exceedingly low numbers and caution against drawing any conclusions for them. All results hold if these languages are included in the final analysis.

2 The averaging is justified as correlations between languages were reasonably high (see results section).

## Data accessibility

All data and code are accessible at the following OSF repository: https://osf.io/w7crs.
